# Dr. Samuel Weissel Gross

**Published:** 1889-05

**Authors:** 


### Obituary.

Dr. Samuel Weissel Gross, Professor of the Principles of Surgery and Clinical Surgery in Jefferson Medical College, died of pneumonia at his home in Philadelphia, Pa., April 16, 1889, aged fifty-two years.

Dr. Gross was a distinguished teacher, author, and clinician, and served in the war of the rebellion as surgeon of U. S. Volunteers, receiving the brevet rank of lieutenant-colonel upon muster-out. He succeeded his renowned father in the chair of surgery upon the retirement of the latter in 1882. His loss to Philadelphia, where he was conspicuous in everything that tends to endear a man to his colleagues, friends, and neighbors, is only greater because nearer to its interests, than to the whole professional world that now bows its head in silent grief in deference to his memory.

